# Effects of compositional heterogeneity and spatial autocorrelation on richness and diversity in simulated landscapes

**DOI:** 10.1002/ece3.10810

**Published:** 2023-12-13

**Authors:** Joseph Tardanico, Thomas Hovestadt

**Affiliations:** ^1^ Department of Animal Ecology and Tropical Biology Julius‐Maximilians Universität Würzburg Germany

**Keywords:** community assembly, diversity, individual based model, landscape structure, metacommunity model, richness

## Abstract

Landscape structure plays a key role in mediating a variety of ecological processes affecting biodiversity patterns; however, its precise effects and the mechanisms underpinning them remain unclear. While the effects of landscape structure have been extensively investigated both empirically and theoretically from a metapopulation perspective, the effects of spatial structure at the landscape scale remain poorly explored from a metacommunity perspective. Here, we attempt to address this gap using a spatially explicit, individual‐based metacommunity model to explore the effects of landscape compositional heterogeneity and per se spatial configuration on diversity at the landscape and patch levels via their influence on long‐term community assembly processes. Our model simulates communities composed of species of annual, asexual organisms living, reproducing, dispersing, and competing within grid‐based, fractal landscapes that vary in their magnitude of spatial environmental heterogeneity and in their degree of spatial environmental autocorrelation. Communities are additionally subject to temporal environmental fluctuations and external immigration, allowing for turnover in community composition. We found that compositional heterogeneity and spatial autocorrelation had differing effects on richness, diversity, and the landscape and patch scales. Landscape‐level diversity was driven by community dissimilarity at the patch level and increased with greater heterogeneity, while landscape richness was largely the result of the short‐term accumulation of immigrants and decreased with greater compositional heterogeneity. Both richness and diversity decreased in variance with greater compositional heterogeneity, indicating a reduction in community turnover over time. Patch‐level richness and diversity patterns appeared to be driven by overall landscape richness and local mass effects, resulting in maximum patch‐level richness and diversity at moderate levels of compositional heterogeneity and high spatial autocorrelation.

## Graphical abstract

The role of landscape spatial structure in shaping biodiversity patterns remains poorly explored from a metacommunity perspective. We used an individual‐based metacommunity model to investigate the effects of compositional heterogeneity and spatial configuration on biodiversity via their effects on community assembly. We show that different degrees of compositional heterogeneity and spatial autocorrelation result in different assembly processes predominating and that richness and diversity are shaped by different processes at the landscape and patch scales, resulting in differing responses to compositional heterogeneity and spatial autocorrelation at different scales.

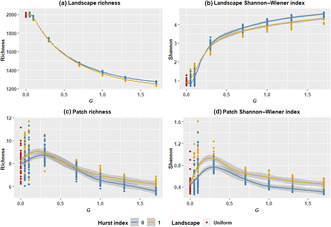

## 1 INTRODUCTION

Land‐use change and habitat destruction remain the principle drivers of biodiversity loss today (Davison et al., 2021; Ellis, 2021; Newbold et al., 2015). A major consequence of the two processes is the alteration of the structure of landscapes by changing the types of environments present in the landscape (composition) and by changing the spatial arrangement of those environment types (configuration; Fahrig, 2003). Landscape structure mediates a large variety of evolutionary and ecological processes such as adaptation and community assembly through its effects on the movement and dispersal of organisms (Fahrig, 2020; Forester et al., 2016; Holt & Barfield, 2011; Tscharntke et al., 2012; Zarnetske et al., 2017) and thus has important implications for biodiversity conservation. A thorough understanding of the role of landscape structure in mediating these processes is therefore critical to developing effective conservation strategies (Rodewald & Arcese, 2016; Sanderson et al., 2002).

Landscape structure has received considerable research attention in recent decades. While greater compositional complexity is expected to result in a larger overall diversity of species (Stein et al., 2014; Tews et al., 2004), the effects of landscape spatial configuration on diversity have been subject to considerable debate (Fahrig et al., 2019; Fletcher Jr. et al., 2018; Hanski, 2015; Semper‐Pascual et al., 2021; Villard & Metzger, 2014). Configurational effects on diversity have typically been investigated in the context of biodiversity conservation in the face of habitat fragmentation. Habitat fragmentation has traditionally been viewed as negative for biodiversity based on island biogeography and metapopulation theory predictions. Habitat fragmentation is expected to reduce species richness by reducing habitat patch size and splitting species populations into smaller, more isolated sub‐populations, thereby increasing the per‐patch risk of extinction and reducing the frequency of recolonization (Hanski, 1998; Hill & Caswell, 1999; MacArthur & Wilson, 1967). The conclusion that habitat fragmentation is generally negative for biodiversity has been challenged on the grounds that many studies on habitat fragmentation measure effects only at the patch scale and do not effectively discriminate between effects resulting from habitat loss and effects of the spatial arrangement itself (fragmentation per se; Arroyo‐Rodríguez et al., 2017; Fahrig, 2003; McGarigal & Cushman, 2002; Tscharntke et al., 2012). Indeed, when total habitat area is controlled for, biodiversity responses to fragmentation are often either non‐significant or positive (Fahrig, 2017; Tscharntke et al., 2012). This apparent lack of a consistent negative effect on species richness has led to the proposal that total habitat amount is the primary determinant of diversity, with configuration playing only a minor role (Fahrig, 2013; Watling et al., 2020). This hypothesis has been disputed on the basis of conflicting empirical evidence (Haddad et al., 2017; Hanski, 2015), as well as due to the lack of a mechanistic explanation (Hanski, 2015). Other authors have pointed out that habitat amount and spatial configuration can interact in a variety of complex ways to influence diversity (Boeye et al., 2014; Püttker et al., 2020; Rybicki et al., 2020; Villard & Metzger, 2014).

While the effect of landscape structure on biodiversity has been extensively investigated empirically, the majority of empirical studies on landscape structure are observational studies, which rely fundamentally on correlative approaches and thus can reveal statistical associations but cannot give direct insight into the causal mechanisms underpinning the patterns they observe (Hanski, 2015; Ovaskainen et al., 2019). Mechanistic modeling approaches have the advantage of allowing direct control over compositional and configurational structure as well as ecological mechanisms, thus permitting detailed experiments that can provide direct insight into causal mechanisms (Cabral et al., 2017; Hanski, 2015; Higgins et al., 2012). Numerous modeling studies have investigated the impacts of landscape structure from a metapopulation perspective (e.g., Hill & Caswell, 1999). These models often explicitly consider spatial structure but only consider populations of a single species. Conclusions regarding community‐level processes drawn from such studies are thus based on extrapolation from the species to the community level. This approach is problematic because different processes acting at different levels of organization can produce counter‐intuitive patterns (McGill, 2019). Metacommunity models consider multiple interacting species, but metacommunity modeling simulation studies investigating biodiversity often do not explicitly consider spatial structure or consider it only in very simplified forms with no explicitly defined spatial geometry (Ai & Ellwood, 2022; Biswas & Wagner, 2012; Ryberg & Fitzgerald, 2016; Zarnetske et al., 2017), while those that do often model landscapes as islands of habitat embedded in a homogeneous, typically uninhabitable matrix (e.g., Firkowski et al., 2022; Thompson et al., 2017). Such an assumption is problematic for terrestrial landscapes, where stark, abrupt shifts in environmental conditions over space are rare and few areas of the landscape can be said to be truly uninhabitable. Indeed, many species exploit multiple habitat types (Hein et al., 2003; Jules & Shahani, 2003), and different species living within the same habitat may vary considerably in their tolerance for environmental variation and thus may have different perceptions of what is habitat and non‐habitat (Prevedello & Vieira, 2010). It may thus be more appropriate in many cases to model landscapes as habitat mosaics or as fractal environmental gradients (Fischer & Lindenmayer, 2006; Franklin & Lindenmayer, 2009; Matthews, 2021), but this is not commonly done (but see Münkemüller et al., 2012).

Here, we attempt to address this gap using a spatially explicit, using an individual‐based metacommunity model implemented in Julia 1.1.1 (Bezanson et al., 2012) based on the model of Sieger and Hovestadt (2020) to systematically explore the effects of landscape structure on patterns of species richness and diversity via their influence on long‐term community assembly processes. Specifically, we ask how varying the strength of compositional heterogeneity and environmental spatial autocorrelation affects patterns of species richness and diversity at the landscape and patch levels. Our model simulates communities composed of species of annual, asexual organisms living, reproducing, dispersing, and competing within continuous grid‐based fractal landscapes, which vary in their magnitude of spatial environmental heterogeneity and in their degree of spatial environmental autocorrelation. Communities are additionally subject to temporal environmental fluctuations and external immigration, allowing for turnover in community composition. Our model produces output data covering the “taxonomic” richness and diversity of simulated organisms, as well as data on organism niches, fitness, and dispersal behavior. This study, however, will focus specifically on results relating to taxonomic richness and diversity.

## 2 MODEL DESCRIPTION

### 2.1 Landscape properties

**FIGURE 1:**
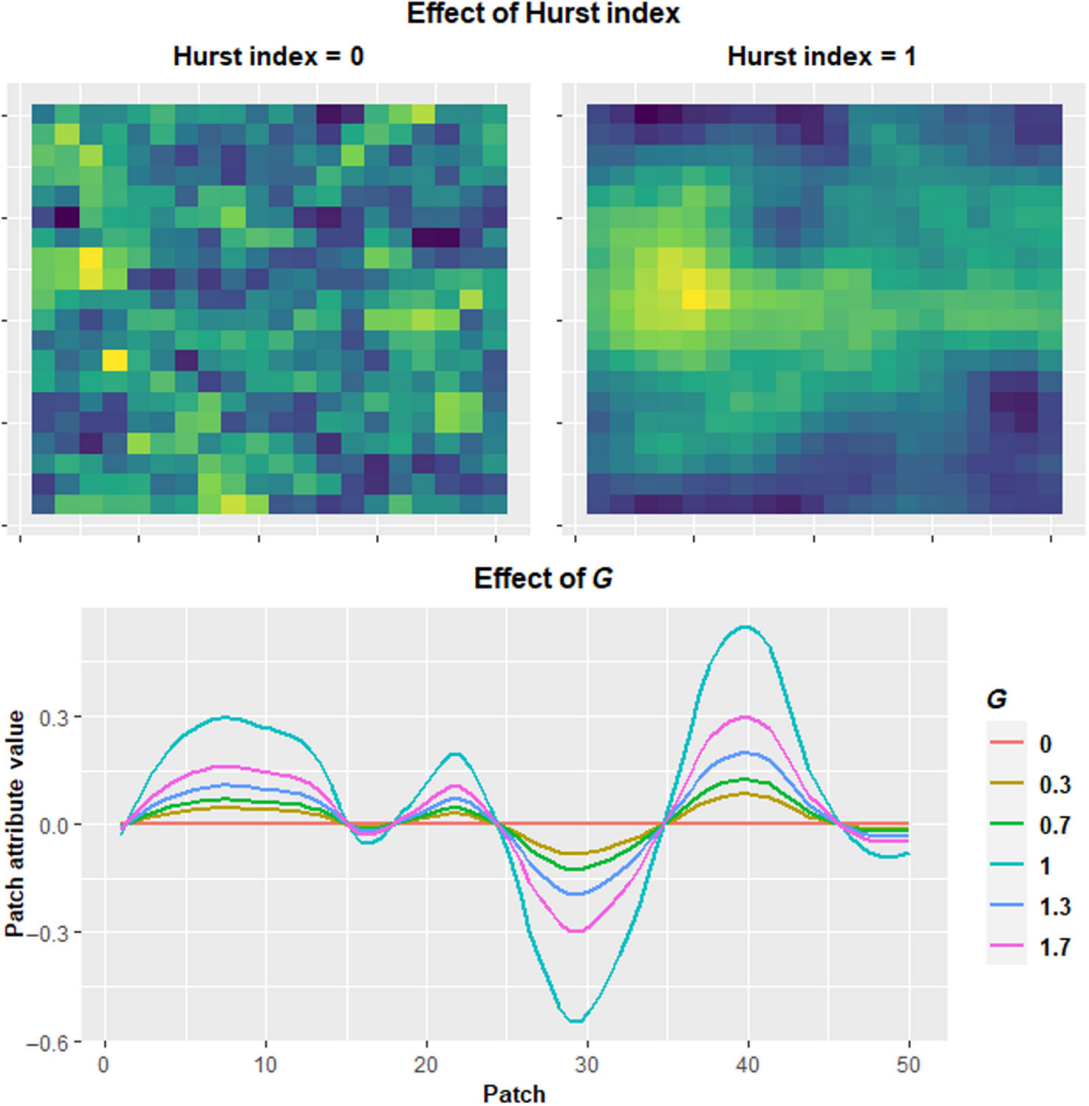
Illustration of the effects of the Hurst index and *G* parameters on landscape structure. The upper panel shows the effect of the Hurst index on spatial autocorrelation. The lower panel shows an example cross section of a landscape for a patch attribute. *G* increases or decreases the range of patch attribute values.

Landscapes consist of grids of habitat patches. Patches possess two attributes: one representing patch temperature (*T*) and a second attribute (*H*) representing an additional, unspecified environmental variable (e.g., a soil property). Spatial distributions for the two patch attributes were generated via an R implementation of the spatially autocorrelated landscape generation algorithm from Saupe (1988). This algorithm is capable of generating fractal landscapes with varying degrees of spatial autocorrelation between grid cell values, depending on the value of the Hurst index parameter. Landscapes generated with this algorithm are toroid, and opposite edges connect seamlessly to each other, thereby preventing edge effects from occurring at landscape edges. In this study, all landscapes were generated with a Hurst index of either 0 or 1. A Hurst index of 1 produces completely spatially autocorrelated landscapes where patches always have similar environments to their immediate neighbors, while a Hurst index of 0 produces a largely random spatial distribution of patch environments (Figure 1). Spatial distributions for the two patch attributes are generated independently, meaning that *T* and *H* attributes do not necessarily correlate with each other spatially. However, *T* and *H* spatial distributions for the same landscape were generated with matching generation parameters, including the Hurst index. Thus, a landscape with a highly autocorrelated spatial distribution for the *T* attribute will always have an equally spatially autocorrelated *H* attribute distribution. Values for patch environmental attributes were drawn from a normal distribution and standardized to a mean of 0 and a standard deviation of 1, such that the average frequency of different patch environment values was constant regardless of spatial configuration. Landscape dimensions were set at 20 by 20 patches, for a total of 400 patches in a landscape. These dimensions were chosen in order to limit computation time while still being large enough for structure‐driven patterns to emerge. Landscape compositional heterogeneity and the magnitude of spatial variation in the *T* and *H* attributes were controlled by the simulation parameter *G*. By multiplying patch attribute values by *G*, the range of values could be expanded or reduced (Figure 1). In addition to varying spatially, the *T* attribute fluctuates over time, such that the *T* attribute for patches varies from one time step to the next. Fluctuations in *T* are global and affect all patches in a landscape equally. Fluctuations in *T* are normally distributed with a mean of 0 and a standard deviation of 1, and patch *T* attributes are modified by adding the value of the fluctuation to the patch's *T* attribute.

### 2.2 Organism properties

Patches are inhabited by populations of asexual organisms belonging to species that behave as a guild of ecologically similar species who compete with each other within a patch. In addition to possessing a “taxonomic” species identity, species possess varying environmental niches and dispersal tendencies, which serve to differentiate species functionally from one another. Organism niches are modeled as Gaussian curves whose center and spread are defined by a niche optimum and tolerance trait, respectively. Organisms possess separate optimum and tolerance traits for *T* and *H*. *T* niche optimum and tolerance are represented by the *T*_opt_ and *T*_tol_ traits, respectively, while *H* niche optimum and tolerance are represented by the *H*_opt_ and *H*_tol_ traits. Organisms also possess two dispersal traits: *P*_disp_, which defines the probability of an organism dispersing from its natal patch, and *P*_global_, which defines an organism's preference for one of two possible dispersal modes. Dispersal is explained further in the section below. Organism traits are summarized in Table 1. Species trait values are generated when a species first appears in a landscape by drawing random values from statistical distributions. Niche optima are drawn from a normal distribution with a *μ* of 0 and *σ* equal to *G*. Tolerance traits are drawn from a log‐normal distribution with a *μ* and *σ* of 0 and 1, respectively. Dispersal traits are drawn from a uniform distribution with a minimum of 0 and a maximum of 1.

**TABLE 1:** Organism traits and initialization distribution parameters.

Trait	Symbol	Distribution	Parameters
Temperature optimum	*T*_opt_	Normal	*μ* = 0, σ = *G*
Temperature tolerance	*T*_tol_	Log‐Normal	*μ* = 0, σ = 1
Habitat optimum	*H*_opt_	Normal	*μ* = 0, σ = *G*
Habitat tolerance	*H*_tol_	Log‐normal	*μ* = 0, σ = 1
Dispersal chance	*P*_disp_	Uniform	0,1
Dispersal mode preference	*P*_global_	Uniform	0,1

### 2.3 Dispersal

Organisms can disperse from their natal patches to other patches. Individual organisms may disperse once during their life cycle. Whether or not an organism disperses from its natal patch is determined by drawing a random number from a uniform distribution and comparing the value with the organism's *P*_disp_ trait. If the random number is less than or equal to the organism's *P*_disp_ trait value, the organism will disperse. Dispersing organisms must then choose a dispersal mode. Two different modes of dispersal are possible within this model, serving as short‐ and long‐distance modes. We chose to explicitly incorporate dispersal distance as a separate trait due to previous research indicating that landscape spatial structure affects dispersal distance differently from dispersal frequency (Gros et al., 2006). Organisms can disperse via nearest‐neighbor dispersal or random global dispersal. We chose these two dispersal methods because they are computationally lightweight, simple to implement, and already in widespread use in modeling studies (Kisdi et al., 2020; Kubisch et al., 2013, 2014; Travis & Dytham, 1999). The dispersal mode is selected by drawing a random number from a uniform distribution between 0 and 1 and comparing its value with an organism's *P*_global_ trait. If the number's value is less than or equal to the organism's *P*_global_ trait, the organism disperses via random global dispersal. If not, the organism disperses via nearest neighbor dispersal. In nearest neighbor dispersal, an organism moves to a random patch with the coordinates *x* + *p* and *y* + *q*, where *x* and *y* are the coordinates for the natal patch and *p* and *q* are integers between −1 and 1. If the target patch's coordinates are outside the bounds of the landscape, the organism is instead moved to the opposite side of the landscape. In random global dispersal, a random patch within the landscape is selected as the target patch. In both dispersal modes, the target patch must have different coordinates from the natal patch and will be re‐selected if the target coordinates leave a dispersing organism in its natal patch.

### 2.4 Organism life cycle

Organisms have annual life cycles with complete replacement of the population at the end of a generation. Life cycles consist of discrete reproduction, competition, and dispersal phases. During the reproductive phase, organisms reproduce asexually to produce offspring with identical traits as their parents. The number of offspring is drawn from a Poisson distribution, with the expected reproductive output determined by an organism's fitness within its patch environment within a given time step as given by Equation 1. Here, *E*_fert_ is the expected number of offspring, *R*_0_ is an organism's intrinsic maximum expected offspring (kept at a constant value of 15), *T*_patch_ and *H*_patch_ are the temperature and habitat values for a given patch. Reproductive output is additionally limited by a trade‐off between tolerance and maximum expected offspring, meaning that organisms with broader tolerances produce fewer offspring on average. This trade‐off serves to prevent organisms from having infinitely large tolerances. The strength of this trade‐off is determined by the trade‐off parameter *α* (Chaianunporn & Hovestadt, 2012; Sieger et al., 2019); lower values produce stronger trade‐offs. As the effect of varying *α* is functionally the same as the effect of varying the strength of *G*, *α* is kept at a constant value of 3 in this study. After reproduction, offspring undergo a maturation phase in which they compete on an equal basis with other offspring within the same patch. Survival of the competition phase is density dependent and regulated via the Beverton‐Holt equations (Equations 2 and 3; Beverton & Holt, 1957), where *S*_*A*_ is the expected surviving offspring, *L*_0_ is the total offspring, and *K* is the carrying capacity of a patch if all organisms in the patch have an *E*_fert_ equal to *R*_0_ and thus perfect fitness. Note that because patch carrying capacity is affected by *E*_fert_, maladaptation may reduce the realized carrying capacity of a patch. The value of *K* is set at 150 individuals, which allows for relatively stable patch populations while maintaining low computation time. The number of surviving offspring are determined by drawing a random number from a binomial distribution with a mean of *S*_*A*_. Surviving offspring are then able to disperse to a new patch and start the cycle anew.

(1) $$E_{\text{fert}}=R_{0}\cdot e^{\frac{-\left(T_{\text{patch}}-T_{\mathrm{opt}}\right)}{2T_{\mathrm{sd}}^{2}}}\cdot e^{\frac{-\left(H_{\text{patch}}-H_{\mathrm{opt}}\right)}{2H_{\mathrm{sd}}^{2}}}\cdot e^{\frac{-T_{\mathrm{sd}}^{2}}{2\alpha^{2}}}\cdot e^{\frac{-H_{\mathrm{sd}}^{2}}{2\alpha^{2}}}$$

(2) $$S_{A}=\frac{1}{1+a\cdot L_{0}}$$

(3) $$a=\frac{R_{0}-1}{K\cdot R_{0}}$$

### 2.5 Immigration from external sources

New organism species can immigrate into the landscape from the outside. The number of new immigrants is randomly drawn from a Poisson distribution with an expected value of *E*_immi_. In our simulations, *E*_immi_ is set at a constant expected value of 2.5 immigrants per patch. This amounts, on average, to approximately 0.0011% of the expected local offspring production for a patch with a perfectly adapted population at carrying capacity. Immigrants are generated with randomized traits within a patch and added to the new generation along with existing offspring. Since immigrants arrive in the landscape from places that may have considerably different environmental conditions, immigrant niche optima are drawn from broader distributions than those used for initialization. The statistical distribution parameters for immigrant traits are summarized in Table 2.

**TABLE 2:** Immigrant trait distributions and parameters.

Trait	Distribution	Parameters
*T*_opt_	Uniform	*μ* = *T*_trend_, *𝜎* = 1.5 * *G*
*T*_tol_	Log‐normal	*μ* = 0, *𝜎* = 1
*H*_opt_	Uniform	*μ* = 0, *𝜎* = 1.5 * *G*
*H*_tol_	Log‐normal	*μ* = 0, *𝜎* = 1
*P*_disp_	Uniform	0,1
*P*_global_	Uniform	0,1

### 2.6 Initialization and experiment design

Landscapes were initialized from text files containing the respective *T* and *H* attribute values for each patch. At initialization, landscapes were uninhabited, and immigrant species were allowed to colonize the landscape over the course of the simulation. Simulations were run for a total of 10,000 steps. Fluctuations in *T* for these time steps were generated at initialization prior to the start of a simulation run. We simulated two Hurst index scenarios (0 and 1) and eight different *G* (compositional heterogeneity) scenarios (*G* ∈ 0,0.05,0.1,0.3,0.7,1,1.3,1.7). We ran each scenario over a set of 10 different landscapes, doing three simulation runs for each landscape, resulting in a total of 30 individual runs for each scenario. The model parameters for our simulations are summarized in Table 3. Simulation runs were initialized with fixed random number generator seeds to ensure replicability. Each individual simulation run in a scenario was initialized with a different starting seed, meaning that replicate runs for the same landscape were not identical. During a simulation run, the simulation program automatically recorded the total population, the total species richness, and calculated the Shannon–Weiner diversity index for the whole landscape at each time step. At time step 10,000, the simulation program recorded a census of every individual organism present in the landscape, recording its location, trait values, and species. The program then used this census data to calculate total population sizes, species richness, and the Shannon–Weiner diversity for each patch in the landscape.

**TABLE 3:** Summary of model parameters.

Parameter	Symbol	Value
Landscape dimensions		20*20 patches
Total simulation time steps	*t*_max_	10,000
Niche breadth trade‐off	*α*	3
Patch Expected immigrants	*E*_immi_	2.5
Gradient strength multiplier	*G*	∈ 0,0.05,0.1,0.3,0.7,1,1.3,1.7
Landscape Hurst Index	*Hurst*	∈ 0,1

### 2.7 Analysis

Data visualization was performed in R 3.6.3 (R Core Team, 2020) using the ggplot2 package (Wickham, 2016). We did not perform any statistical significance tests, as such tests are not useful or meaningful in the context of mechanistic modeling due to the extreme sensitivity of such tests to small differences when used with very large numbers of observations (White et al., 2014). Instead, results were assessed visually via box plots and scatter plots with GAM smoother curves using the Gaussian family with an identity link and a k of 7. Curves were calculated using data aggregated by calculating mean values for each combination of *G*, Hurst index, landscape, and replicate to reduce computation time. We assessed landscape‐level patterns via the distribution of landscape richness and Shannon–Weiner diversity between the 5000th and 10,000th time steps and patch‐level patterns via the distribution of patch richness and Shannon–Weiner diversity at the end of the simulation.

## 3 RESULTS

**FIGURE 2:**
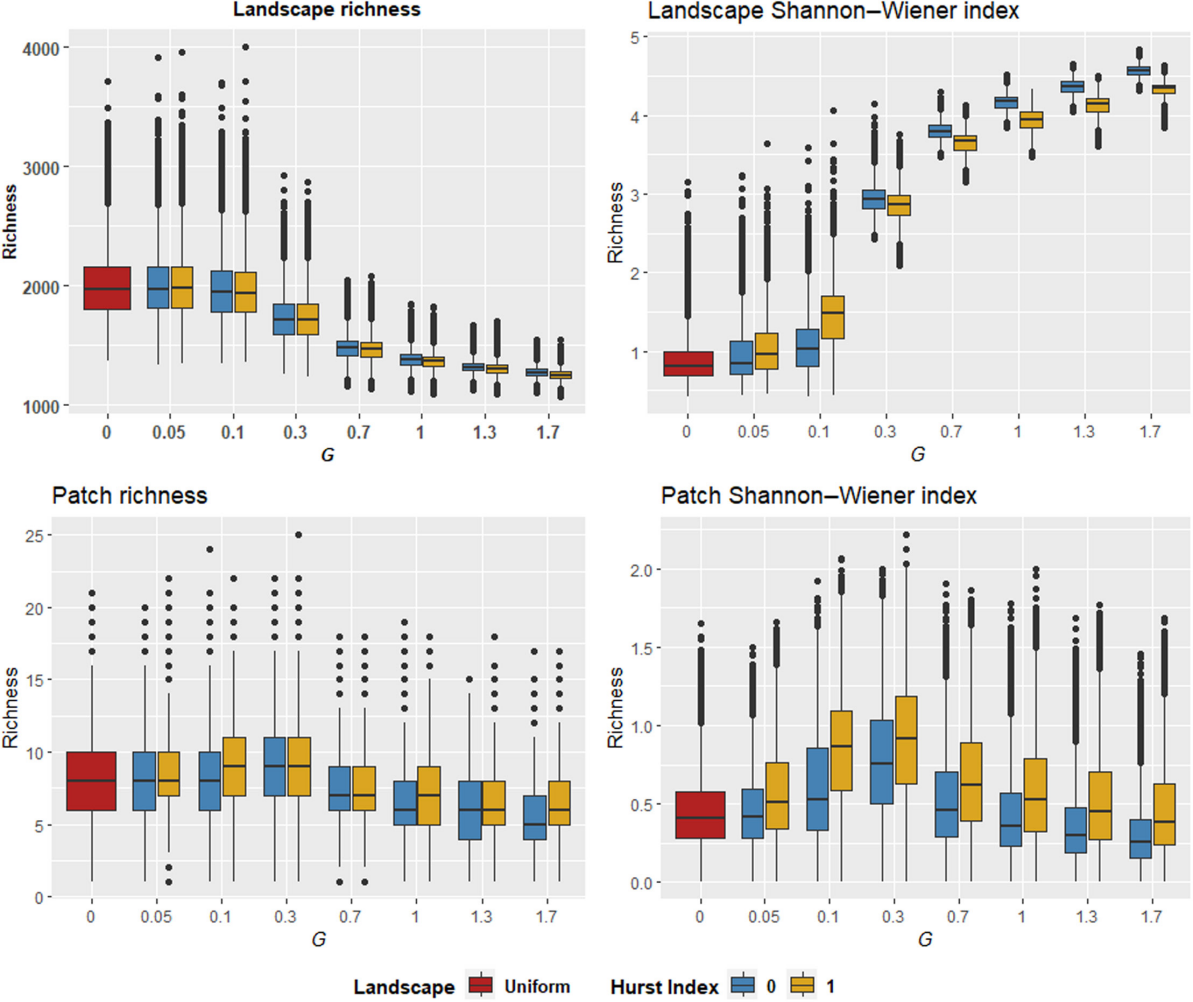
Box plots of richness and Shannon diversity by *G* and Hurst index scenario. Landscape‐level richness and diversity distributions are shown for time steps 5000–10,000. Patch distributions are shown for time step 10,000.

**FIGURE 3:**
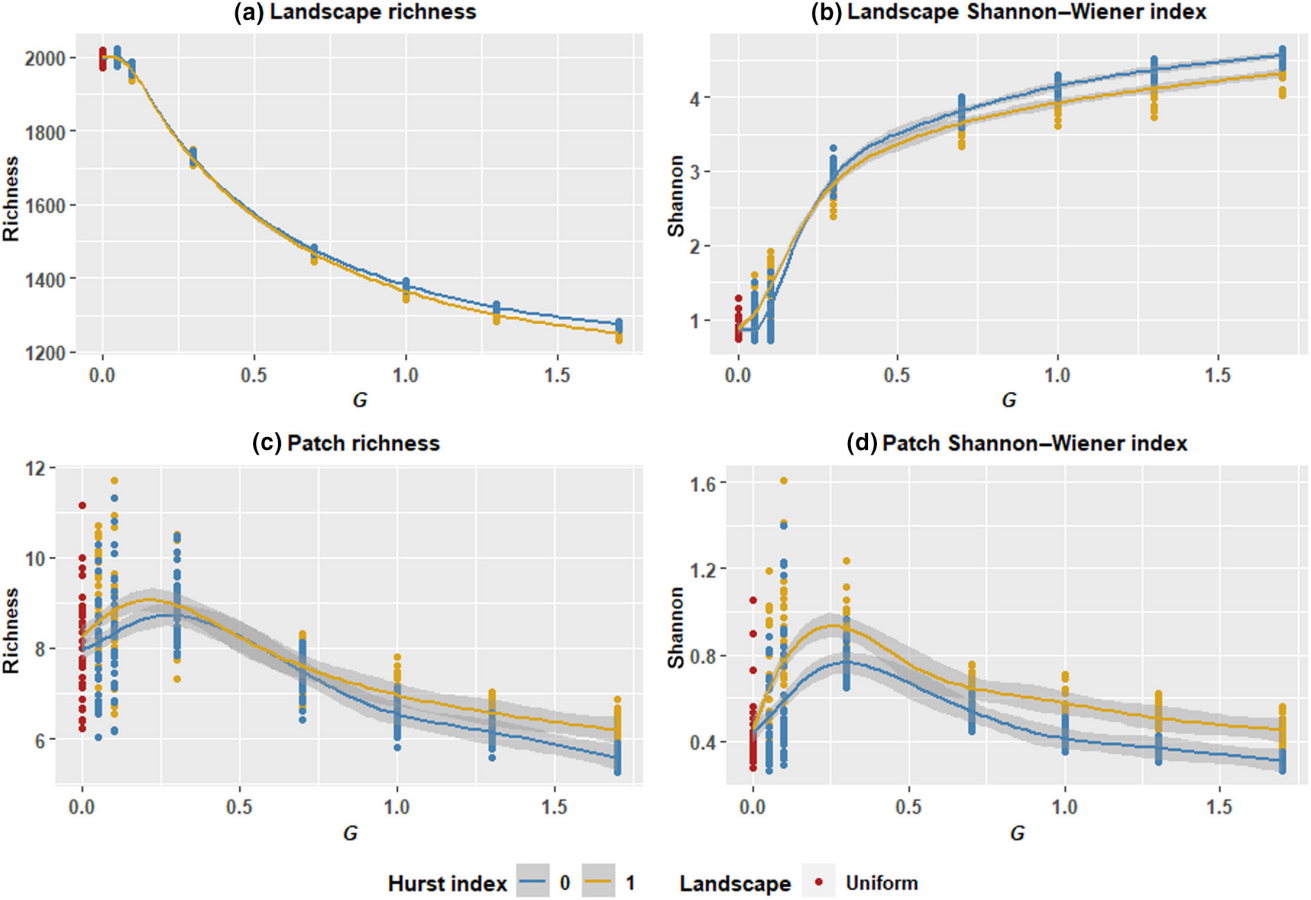
Scatterplots for richness and Shannon–Wiener diversity at the landscape and patch levels with GAM curves. Curves were calculated using data aggregated by taking the mean for each combination of *G*, Hurst index, landscape, and replicate (*n* = 450). Gaussian family with identity link (*k* = 7).

The landscape total population rapidly increases within the first 50 time steps, settling into a stable median slightly below the landscape carrying capacity of 60,000 individuals (150 individuals per patch), around which it fluctuates. Species richness and Shannon–Wiener diversity index responded at both the landscape and patch levels to both compositional heterogeneity and spatial autocorrelation, with differing responses occurring at the landscape and patch levels (Figures 2 and 3). At the landscape level, median richness and Shannon–Wiener diversity show roughly log‐sigmoidal, opposing relationships with *G*, exhibiting initially strong responses beyond a threshold that diminish toward higher values of *G*, while variance for the two declined with greater *G*. Landscape median richness decreased with increasing *G* starting at *G* = 0.1, declining relatively steeply at first with diminishing declines beyond *G* = 0.7. From *G* = 0.7 onwards, a slight difference emerges between Hurst index scenarios, with Hurst index = 0 scenarios showing higher landscape richness. Landscape Shannon–Wiener diversity showed an opposing pattern, increasing in median value with greater *G*, rapidly at first, with increases diminishing beyond *G* = 0.7. The precise relationship between landscape diversity and *G* was affected by spatial autocorrelation. Below *G* = 0.1, landscape diversity was higher in Hurst index = 1 scenarios, while above this threshold, landscape diversity was higher in Hurst index = 0 scenarios. Patch richness and diversity were unimodal in relation to *G*, peaking at *G* = 0.3 and gradually declining thereafter. This pattern was notably pronounced for patch diversity, while for patch richness, the response to *G* was more muted. Median patch Shannon–Wiener diversity was consistently higher in Hurst index = 1 scenarios, while a similar though less consistent pattern occurred with patch richness.

## 4 DISCUSSION

Our simulation results showed that compositional heterogeneity (*G*) and spatial autocorrelation (Hurst index) had differing effects at the landscape and patch levels, resulting from different processes acting at different scales. Landscape‐level diversity in this model resulted from community dissimilarity at the patch level due to greater environmental heterogeneity and a more fragmented spatial configuration. This finding aligns with the predictions of the dominance of diversity hypothesis (Tscharntke et al., 2012) and with empirical studies finding heterogeneity‐driven diversity to be an important driver of landscape‐scale diversity (Clough et al., 2007; Kessler et al., 2009; Quinn & Harrison, 1988; Tscharntke et al., 2002; Wintle et al., 2019). The increase in diversity occurred despite a pattern of decreasing landscape‐level richness in relation to environmental heterogeneity. Likewise, we found different patterns for landscape‐level and patch‐level richness and diversity, similar to previous empirical research documenting contrasting biodiversity patterns at different scales (Chase & Leibold, 2002; Flohre et al., 2011; Gao et al., 2021; Hendrickx et al., 2007; Tello et al., 2015). Richness and diversity patterns at the patch level appear to be primarily related to overall landscape richness (Tscharntke et al., 2012) and the strength of mass effects resulting from spillover from neighboring patches (Leibold et al., 2004). Our model demonstrates that these patterns and processes can result from the combined effects of spatial autocorrelation and increasing subdivision of the landscape among different environments on rates of successful colonization and extinction. These findings have potentially important implications for biodiversity conservation as they suggest that there may be a trade‐off in optimizing the design of biodiversity reserves for diversity at the patch versus landscape level.

Increasing the compositional heterogeneity of a landscape increases the number of distinct environmental conditions in the landscape. The increasing range of environments in the landscape increases the number of environment types available for organisms to exploit while also imposing increasing fitness costs for generalist organisms and increasing the risks of dispersal (Hastings, 1983). Increasing compositional heterogeneity thus results in increasingly distinct local communities and more local competition. This effect can be further compounded by highly fragmented spatial configurations, which may restrict organisms from colonizing all of their available habitat (Bascompte & Solé, 1996; Hill & Caswell, 1999; McInerny et al., 2007) and prevent competitors from interacting with each other (Boeye et al., 2014), thus slowing or preventing competitive exclusion. Taken together, these effects may account for the higher diversity and the tendency toward slightly higher richness in landscapes with low spatial autocorrelation, as well as the lower patch‐level diversity and the tendency toward lower patch richness in highly heterogeneous landscapes. Conversely, extremely homogeneous landscapes have only a narrow range of exploitable habitats and impose little cost on dispersal, resulting in intense, landscape‐wide competition and producing homogeneous landscape communities dominated by a small number of highly competitive organisms. Meanwhile, moderate compositional heterogeneity allows for the formation of distinct local communities dominated by locally adapted organisms, but also permits enough dispersal for the emergence of source‐sink dynamics leading to mass effects Leibold et al. (2004), which would explain why patch‐level richness and diversity peak at moderate levels of heterogeneity.

The increasing subdivision of the landscape among different environmental types also appears to affect the establishment and survival of immigrant populations. In homogeneous landscapes, a certain portion of immigrants will be highly likely to establish populations due to the large number of suitable patches in the landscape. However, most immigrant populations will remain small due to intense global competition with abundant, competitively dominant organisms. The small size of immigrant populations and the lack of bet‐hedging opportunities in homogeneous landscapes render these populations vulnerable to stochastic or disturbance‐induced extinction (Hanski, 1998; Lande, 1993), which may explain why variance in landscape richness and diversity is greater in more homogeneous landscapes. Homogenous landscapes can accumulate large numbers of species in the short term, but they can also lose them very suddenly due to extreme environmental fluctuations. In highly heterogeneous landscapes, immigrants are unlikely to land in a suitable patch, but those that do face limited, local competition are thus able to achieve larger population sizes on average than immigrants in homogeneous landscapes. This results in a landscape community that is less rich overall but also less globally dominated by a small number of highly abundant species. Due to their larger population sizes and the bet‐hedging opportunities afforded by a spatially heterogeneous environment, these populations are likely to survive extreme environmental fluctuations (Sieger & Hovestadt, 2020), resulting in a more stable landscape community composition in the long term.

As with all models, our model makes a number of simplifying assumptions. Organisms in this model possess annual life cycles with no overlap between generations. The inclusion of longer‐lived, iteroparous organisms would alter model dynamics due to the additional bet‐hedging options such organisms have against temporal environmental variation, as reproduction can be spread out over time or timed to maximize their offspring's chances of survival (Danforth, 1999; Gremer & Venable, 2014; Hopper, 1999), resulting in a lower extinction rate and slower turnover in community composition. In a model with competing annual and perennial organisms, this should result in a pattern of succession ending with long‐lived organisms dominating the landscape. On the other hand, the slower population turnover in long‐lived organisms may slow the process of adaptation to changing conditions and result in extinction debts (Hylander & Ehrlén, 2013). Similarly, dormancy can serve to increase population persistence by serving as a sort of “dispersal through time” (Buoro & Carlson, 2014), allowing organisms to spread out risk temporally or avoid unfavorable conditions, which could increase landscape‐level diversity. Our study did not consider the effects of mutation on richness and diversity. Mutations affecting niche traits may allow a species to expand its effective niche by diversifying individual niche traits, which could result in a species preempting habitat that would otherwise be colonized by another species, likely resulting in reduced landscape richness and diversity. The effect of mutation would likely be most apparent under low to moderate compositional heterogeneity, where there is sufficient spatial environmental variation to create distinct zones that not all organisms can survive in but where heterogeneity is not strong enough to harshly penalize dispersal. Model results may also be affected by the dispersal strategies employed by organisms. Organisms in this model are limited to two modes of dispersal, both of which are undirected and independent of population density or other local conditions. Undirected, state‐ and fitness‐independent dispersal carries a significant risk that a dispersing organism will end up in an unsuitable habitat or disperse at an inopportune time. As such, fitness, and therefore population persistence, will be highly sensitive to spatial context. Informed and directed dispersal has the potential to greatly reduce the risks of dispersal (Lakovic et al., 2015), enabling more frequent dispersal in otherwise high‐risk spatial contexts (Sieger & Hovestadt, 2021). Directed long‐distance dispersal could improve population persistence in the landscape by facilitating colonization of otherwise isolated patches, but it could also reduce β‐diversity by allowing competitively dominant species to spread to suitable habitat more easily in more fragmented landscapes (Catano et al., 2017; Grainger & Gilbert, 2016). Biotic interactions such as mutualisms, facilitation, and trophic interactions also have the potential to shape biodiversity patterns in a variety of complex ways (McIntire & Fajardo, 2014; Mod et al., 2016; Sandor et al., 2022; Wardle, 2006), but only competition is considered in this study.

## 5 CONCLUSIONS

Our study demonstrates the important role played by both compositional and configurational landscape structure in shaping community assembly processes, something that has previously received little attention in metacommunity simulation studies. Our model reproduced several patterns documented by previous empirical studies or predicted by theoretical research, all arising as a result of the effects of landscape structure on colonization, dispersal, and extinction rates. We found that different processes can dominate at different scales, leading to different relationships between richness, diversity, and landscape structure at the landscape and patch levels. Our findings thus carry potentially significant implications for the design of biodiversity reserves as they suggest conservation trade‐offs between different spatial structures at different spatial scales. Given the ongoing biodiversity crisis, there is an urgent need for additional research on the mechanisms underpinning spatial biodiversity patterns. Future studies should consider the roles of additional processes such as trophic interactions and mutualisms, as well as potential interactions with climate change.

## AUTHOR CONTRIBUTIONS

**Joseph Tardanico:** Conceptualization (equal); data curation (lead); investigation (lead); methodology (lead); software (lead); visualization (lead); writing – original draft (lead). **Thomas Hovestadt:** Conceptualization (equal); funding acquisition (lead); investigation (supporting); methodology (supporting); project administration (lead); supervision (lead); visualization (supporting); writing – review and editing (supporting).

## FUNDING INFORMATION

This study was conducted within the framework of the joint project Landklif (https://www.landklif.biozentrum.uni‐wuerzburg.de/) funded by the Bavarian Ministry of Science and the Arts via the Bavarian Climate Research Network (Bayklif). This publication was supported by the Open Access Publication Fund of the University of Würzburg.

## Supporting information


Data S1.

Data S2.

## Data Availability

Simulation output data used in this manuscript, as well as configuration files and shell scripts used to run the simulations and the R scripts used for analysis, are archived on the Dryad Digital Repository: doi:10.5061/dryad.mcvdnck5d. Code for the simulation program used to generate the data is available from GitHub: https://github.com/jtardanico/TardanicoHovestadt2023_Landscapes.
