# Supplementary material for: Effects of compositional heterogeneity and spatial autocorrelation on richness and diversity in simulated landscapes
Source: Ecol Evol. 2023 Dec 13;13(12):e10810. doi: 10.1002/ece3.10810 (PMC10716673; doi:10.1002/ece3.10810)
Supplement: Supplementary file 1 — Data S1. [file ECE3-13-e10810-s002.pdf]

Comparison of original and new simulation output data

Shell script code for original simulation runs. -t and -e inputs specify T and H attributes respectively for patches, defining the landscape. -r inputs specify seeds for random number generation. Only 10 landscapes were used, but with 30 different seeds. Simulations should have had a unique landscape for each replicate run.

```
./julia -p 1 $source -n $para -t $T1  -e $H1 -r 1
./julia -p 1 $source -n $para -t $T2  -e $H2 -r 2
./julia -p 1 $source -n $para -t $T3  -e $H3 -r 3
./julia -p 1 $source -n $para -t $T4  -e $H4 -r 4
./julia -p 1 $source -n $para -t $T5  -e $H5 -r 5
./julia -p 1 $source -n $para -t $T6  -e $H6 -r 6
./julia -p 1 $source -n $para -t $T7  -e $H7 -r 7
./julia -p 1 $source -n $para -t $T8  -e $H8 -r 8
./julia -p 1 $source -n $para -t $T9  -e $H9 -r 9
./julia -p 1 $source -n $para -t $T10  -e $H10 -r 10

./julia -p 1 $source -n $para -t $T1  -e $H1 -r 11
./julia -p 1 $source -n $para -t $T2  -e $H2 -r 12
./julia -p 1 $source -n $para -t $T3  -e $H3 -r 13
./julia -p 1 $source -n $para -t $T4  -e $H4 -r 14
./julia -p 1 $source -n $para -t $T5  -e $H5 -r 15
./julia -p 1 $source -n $para -t $T6  -e $H6 -r 16
./julia -p 1 $source -n $para -t $T7  -e $H7 -r 17
./julia -p 1 $source -n $para -t $T8  -e $H8 -r 18
./julia -p 1 $source -n $para -t $T9  -e $H9 -r 19
./julia -p 1 $source -n $para -t $T10  -e $H10 -r 20

./julia -p 1 $source -n $para -t $T1  -e $H1 -r 21
./julia -p 1 $source -n $para -t $T2  -e $H2 -r 22
./julia -p 1 $source -n $para -t $T3  -e $H3 -r 23
./julia -p 1 $source -n $para -t $T4  -e $H4 -r 24
./julia -p 1 $source -n $para -t $T5  -e $H5 -r 25
./julia -p 1 $source -n $para -t $T6  -e $H6 -r 26
./julia -p 1 $source -n $para -t $T7  -e $H7 -r 27
./julia -p 1 $source -n $para -t $T8  -e $H8 -r 28
./julia -p 1 $source -n $para -t $T9  -e $H9 -r 29
./julia -p 1 $source -n $para -t $T10  -e $H10 -r 30
```

Corrected shell script. Each replicate run uses a different landscape

```
./julia -p 1 $source -n $para -t $T1  -e $H1 -r 1
./julia -p 1 $source -n $para -t $T2  -e $H2 -r 2
./julia -p 1 $source -n $para -t $T3  -e $H3 -r 3
./julia -p 1 $source -n $para -t $T4  -e $H4 -r 4
./julia -p 1 $source -n $para -t $T5  -e $H5 -r 5
./julia -p 1 $source -n $para -t $T6  -e $H6 -r 6
./julia -p 1 $source -n $para -t $T7  -e $H7 -r 7
./julia -p 1 $source -n $para -t $T8  -e $H8 -r 8
./julia -p 1 $source -n $para -t $T9  -e $H9 -r 9
./julia -p 1 $source -n $para -t $T10  -e $H10 -r 10

./julia -p 1 $source -n $para -t $T11  -e $H11 -r 11
./julia -p 1 $source -n $para -t $T12  -e $H12 -r 12
./julia -p 1 $source -n $para -t $T13  -e $H13 -r 13
./julia -p 1 $source -n $para -t $T14  -e $H14 -r 14
./julia -p 1 $source -n $para -t $T15  -e $H15 -r 15
./julia -p 1 $source -n $para -t $T16  -e $H16 -r 16
./julia -p 1 $source -n $para -t $T17  -e $H17 -r 17
./julia -p 1 $source -n $para -t $T18  -e $H18 -r 18
./julia -p 1 $source -n $para -t $T19  -e $H19 -r 19
./julia -p 1 $source -n $para -t $T20  -e $H20 -r 20

./julia -p 1 $source -n $para -t $T21  -e $H21 -r 21
./julia -p 1 $source -n $para -t $T22  -e $H22 -r 22
./julia -p 1 $source -n $para -t $T23  -e $H23 -r 23
./julia -p 1 $source -n $para -t $T24  -e $H24 -r 24
./julia -p 1 $source -n $para -t $T25  -e $H25 -r 25
./julia -p 1 $source -n $para -t $T26  -e $H26 -r 26
./julia -p 1 $source -n $para -t $T27  -e $H27 -r 27
./julia -p 1 $source -n $para -t $T28  -e $H28 -r 28
./julia -p 1 $source -n $para -t $T29  -e $H29 -r 29
./julia -p 1 $source -n $para -t $T30  -e $H30 -r 30
```

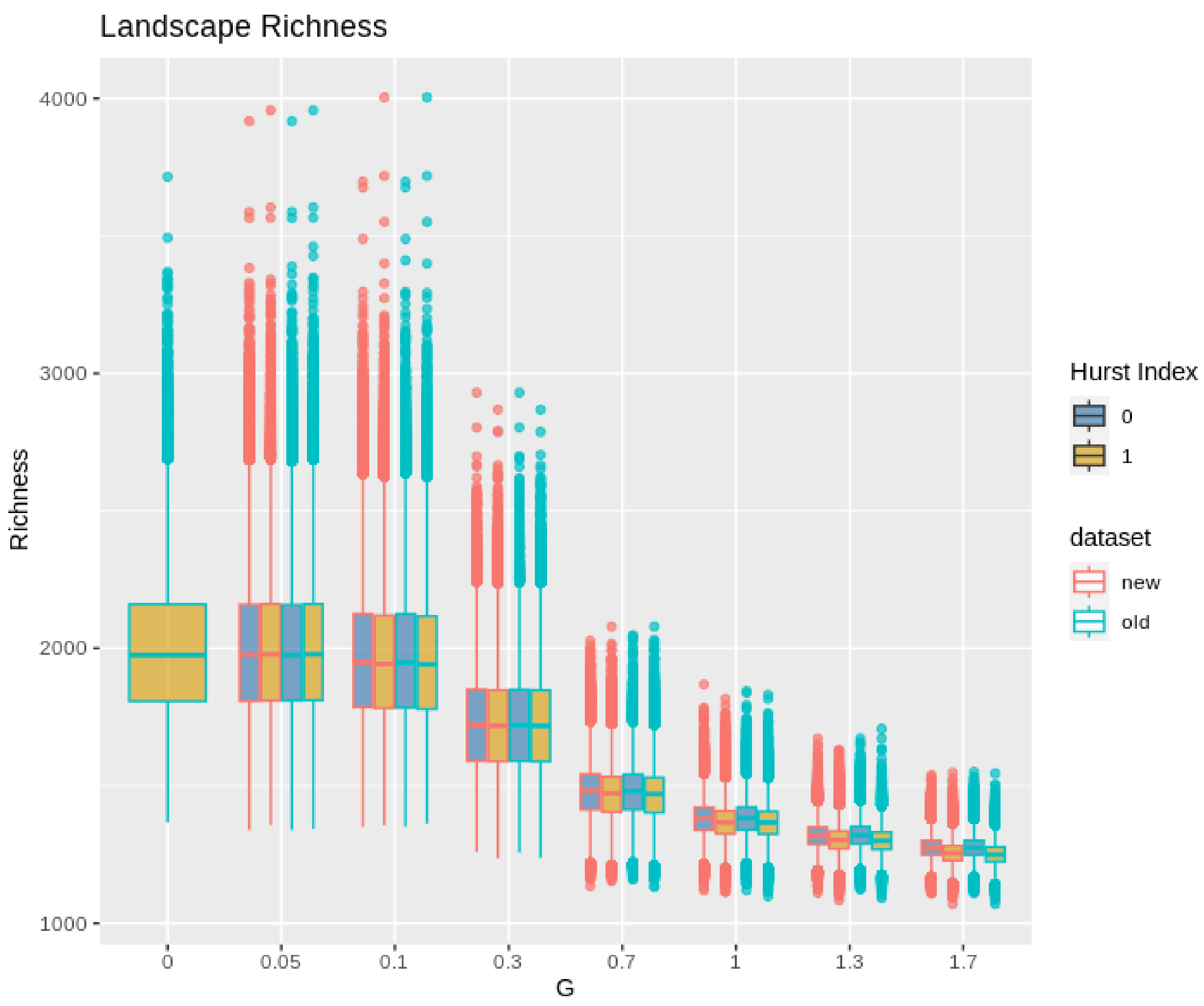

Box plots of landscape richness by dataset, G and Hurst index scenario. Landscape level richness and diversity distributions are shown for time steps 5000 to 10000.

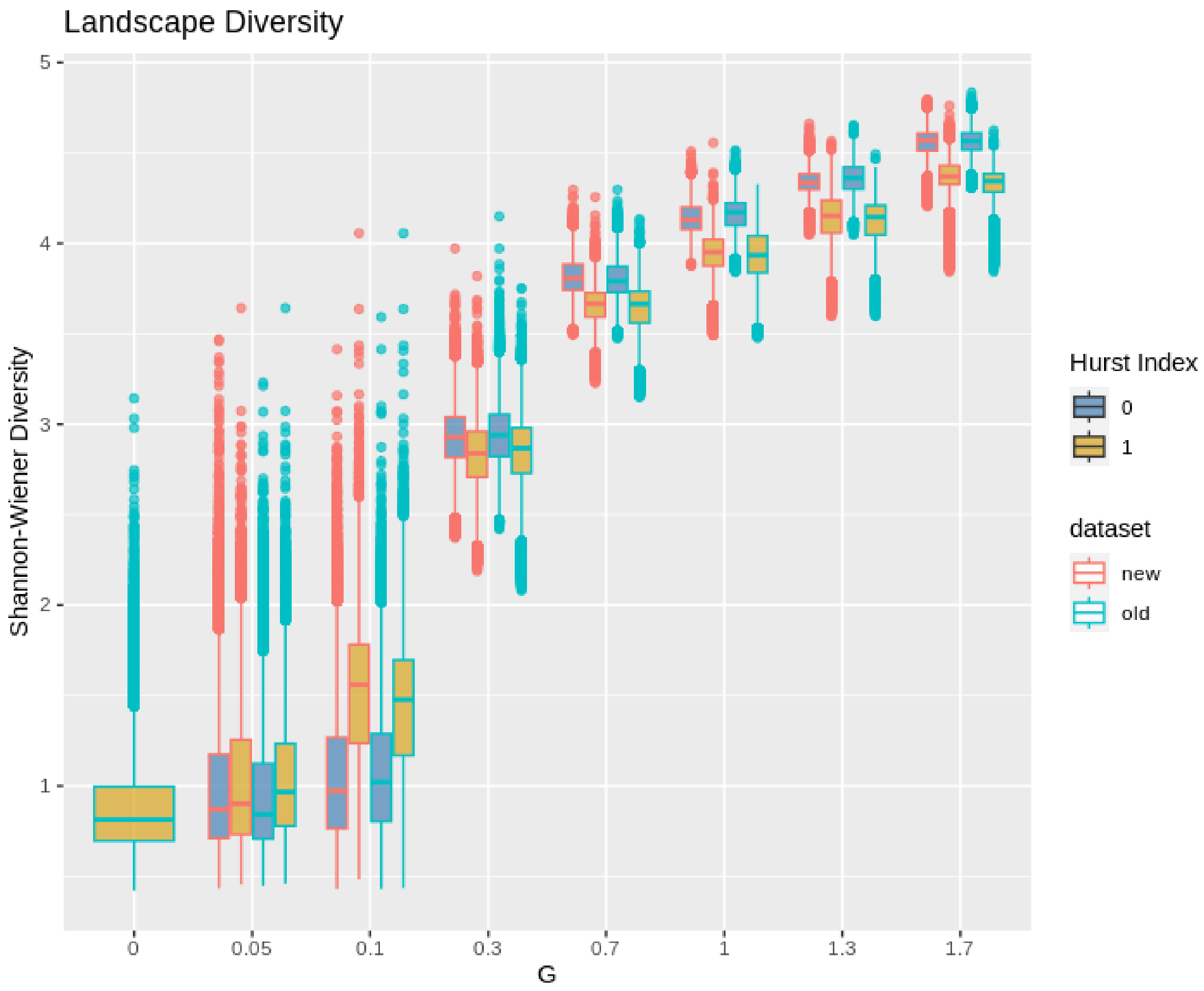

Box plots of landscape Shannon-Wiener diversity index by dataset, G and Hurst index scenario. Landscape level richness and diversity distributions are shown for time steps 5000 to 10000.

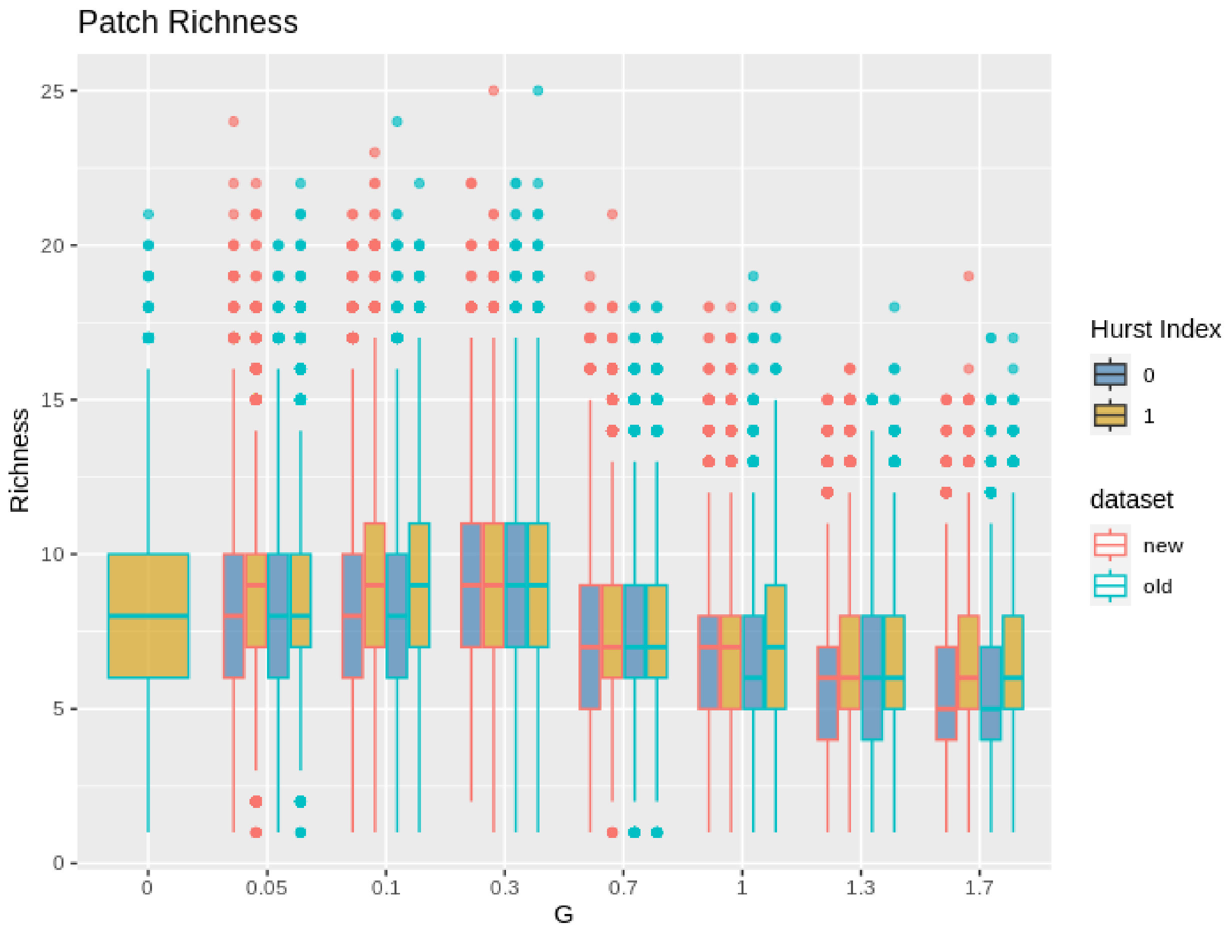

Box plots of patch richness by dataset, G and Hurst index scenario. Distributions are shown for time step 10000.

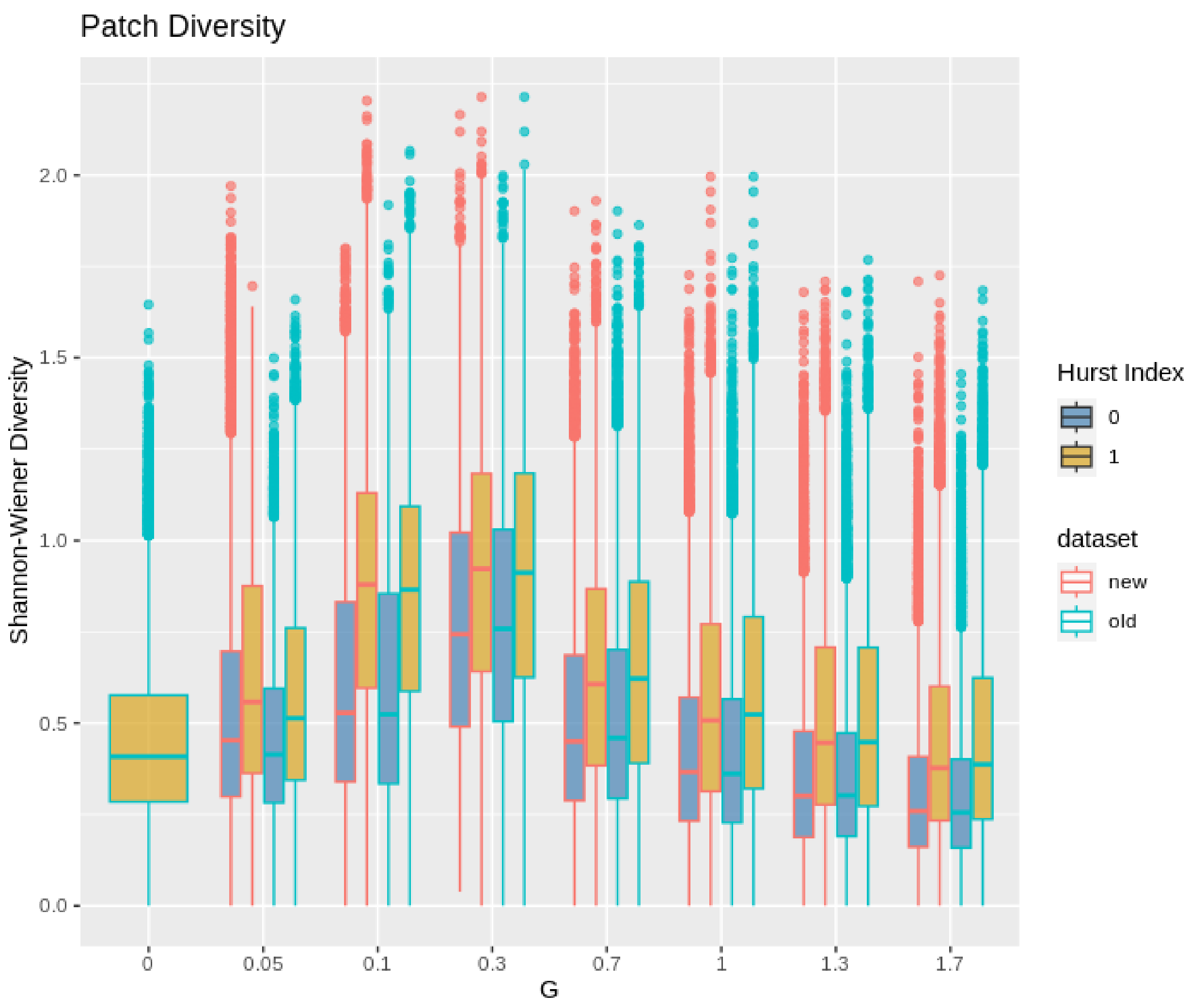

Box plots of patch Shannon-Wiener diversity index by dataset, G and Hurst index scenario. Distributions are shown for time step 10000.
