# Supplementary material for: Effects of compositional heterogeneity and spatial autocorrelation on richness and diversity in simulated landscapes
Source: Ecol Evol. 2023 Dec 13;13(12):e10810. doi: 10.1002/ece3.10810 (PMC10716673; doi:10.1002/ece3.10810)
Supplement: Supplementary file 2 — Data S2. [file ECE3-13-e10810-s001.pdf]

Supplememtary Materials: Effects of compositional heterogeneity and spatial autocorrelation on richness and diversity in simulated landscapes

Joseph Tardanico

Thomas Hovestadt

Supplementary Table S1. Summary of GAM model output. Models were run over data aggregated by taking the mean for each combination of G, Hurst index, landscape, and replicate (n=450) to reduce computation time. Gaussian family with identity link (k=7).

Model	Parameter	Coef	SE	CI Lower	CI Upper
Landscape richness	Intercept	1642.41	13.59	1615.78	1669.04
	Hurst_index	0.00	0.00	0.00	0.00
Landscape diversity	Intercept	2.84	0.07	2.70	2.98
	Hurst_index	0.00	0.00	0.00	0.00
Patch richness	Intercept	7.36	0.07	7.22	7.51
	Hurst_index	0.00	0.00	0.00	0.00
Patch diversity	Intercept	0.49	0.01	0.47	0.52
	Hurst_index	0.00	0.00	0.00	0.00

Model	Parameter (edf)
Landscape richness	s(G):Hurst_index (4.17)
Landscape diversity	s(G):Hurst_index (4.83)
Patch richness	s(G):Hurst_index (5.50)
Patch diversity	s(G):Hurst_index (6.66)

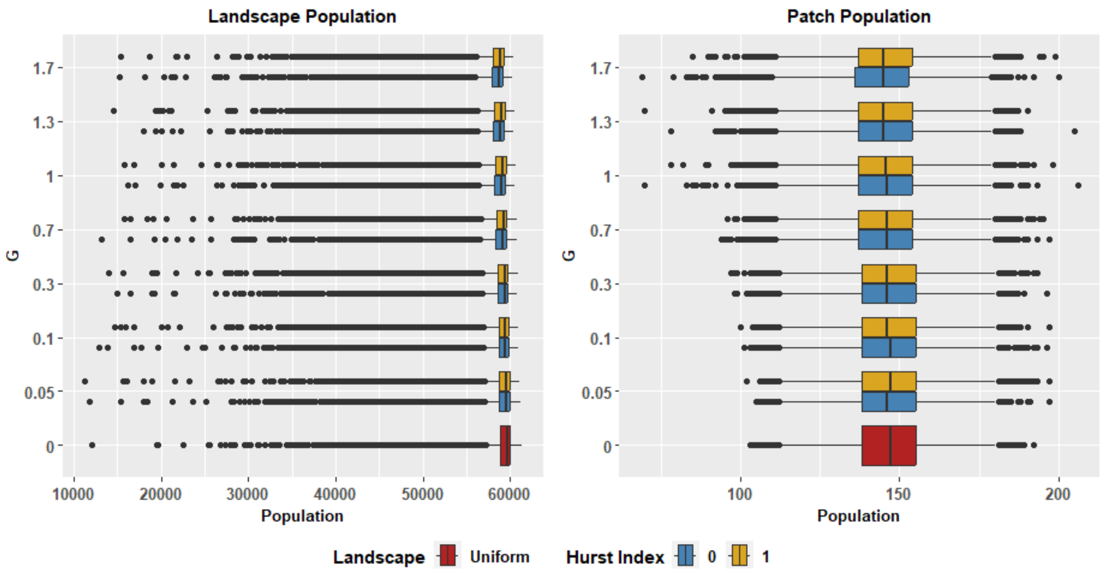

Supplementary Figure S1. Box plots of landscape and patch level total population by G and Hurst index scenario. Landscape population distributions are shown for time steps 5000 to 10000. Patch distributions are shown for time step 10000.

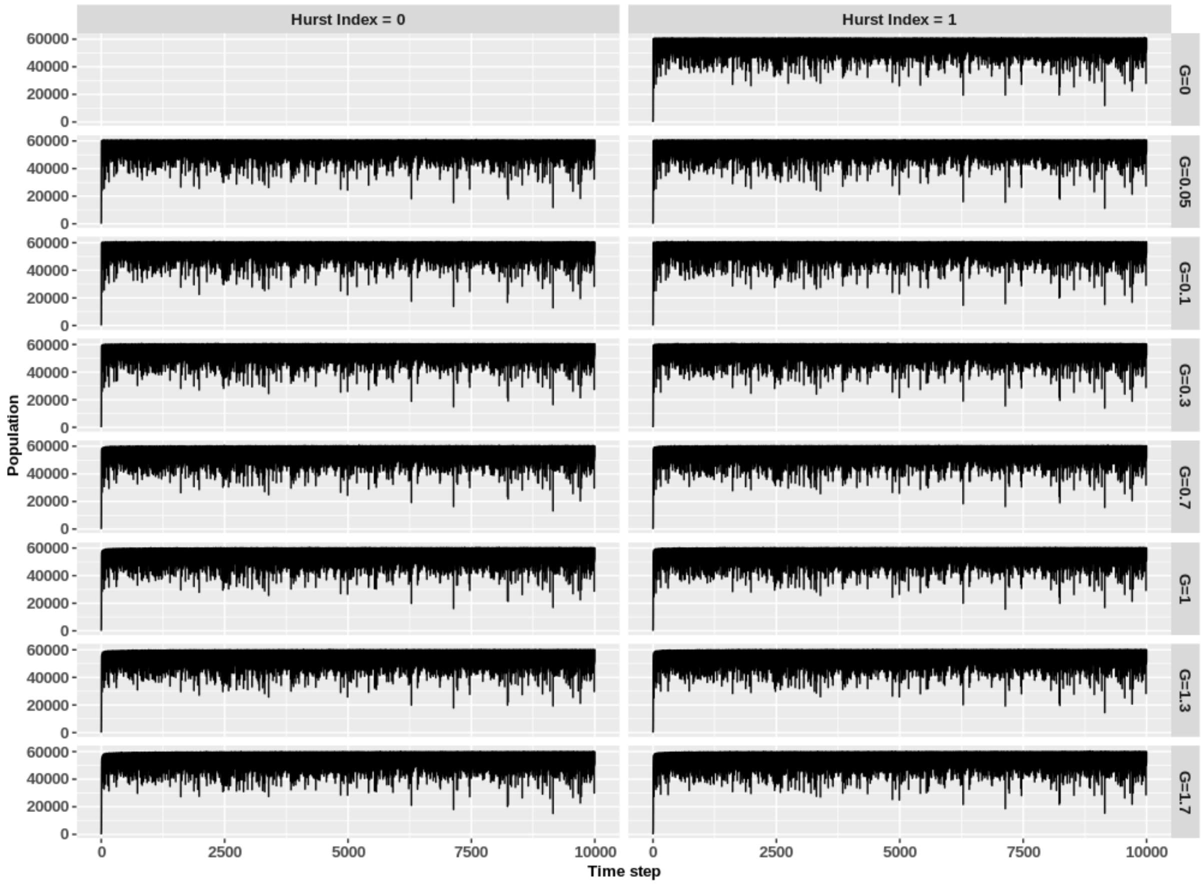

Supplementary Figure S2. Landscape total population over time

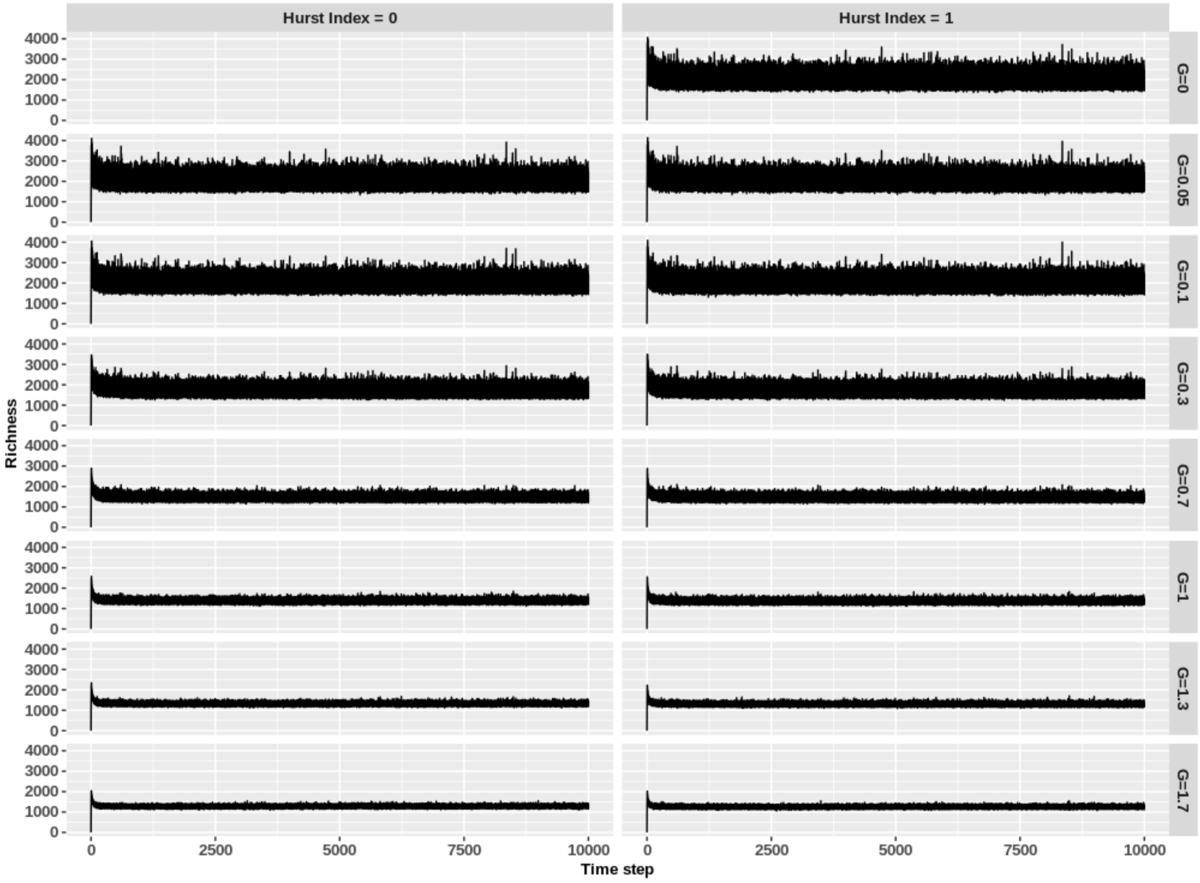

Supplementary Figure S3. Landscape richness over time

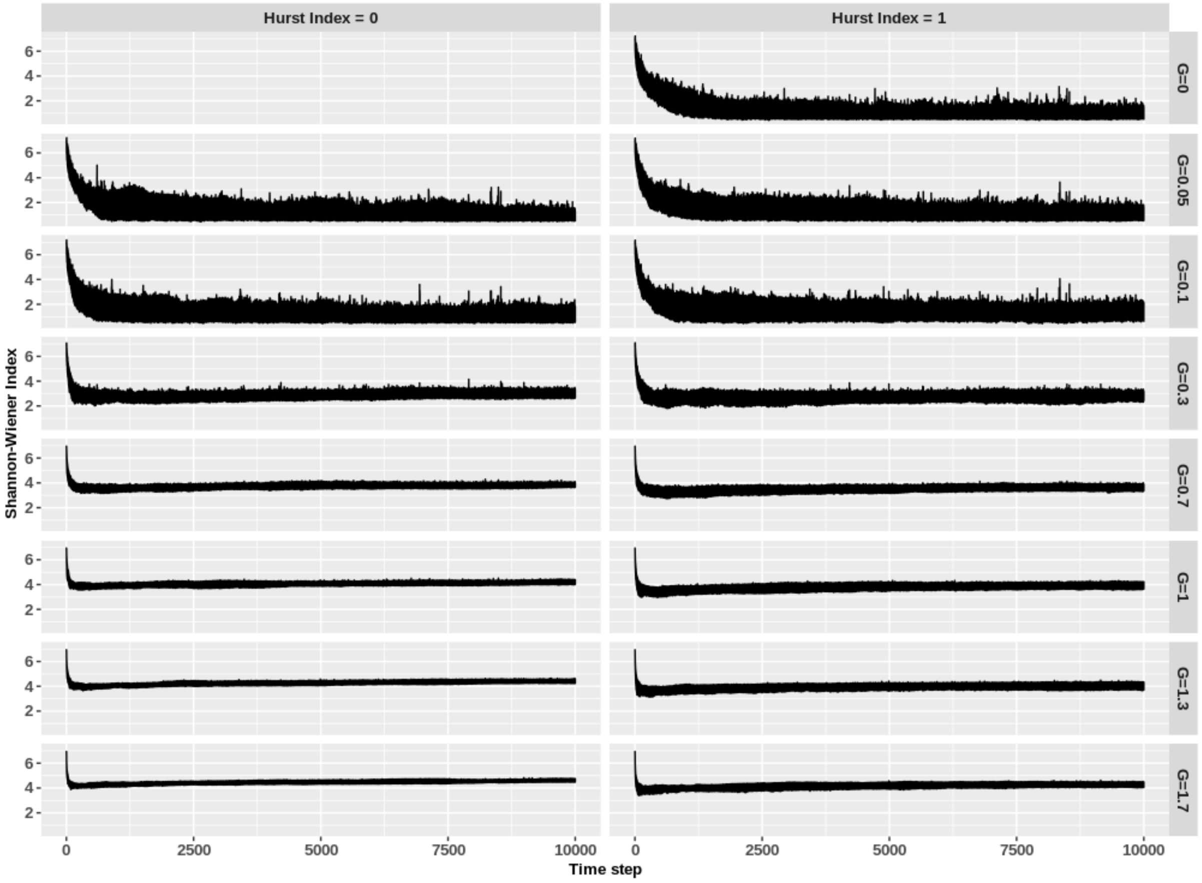

Supplementary Figure S4. Landscape Shannon-Wiener index over time

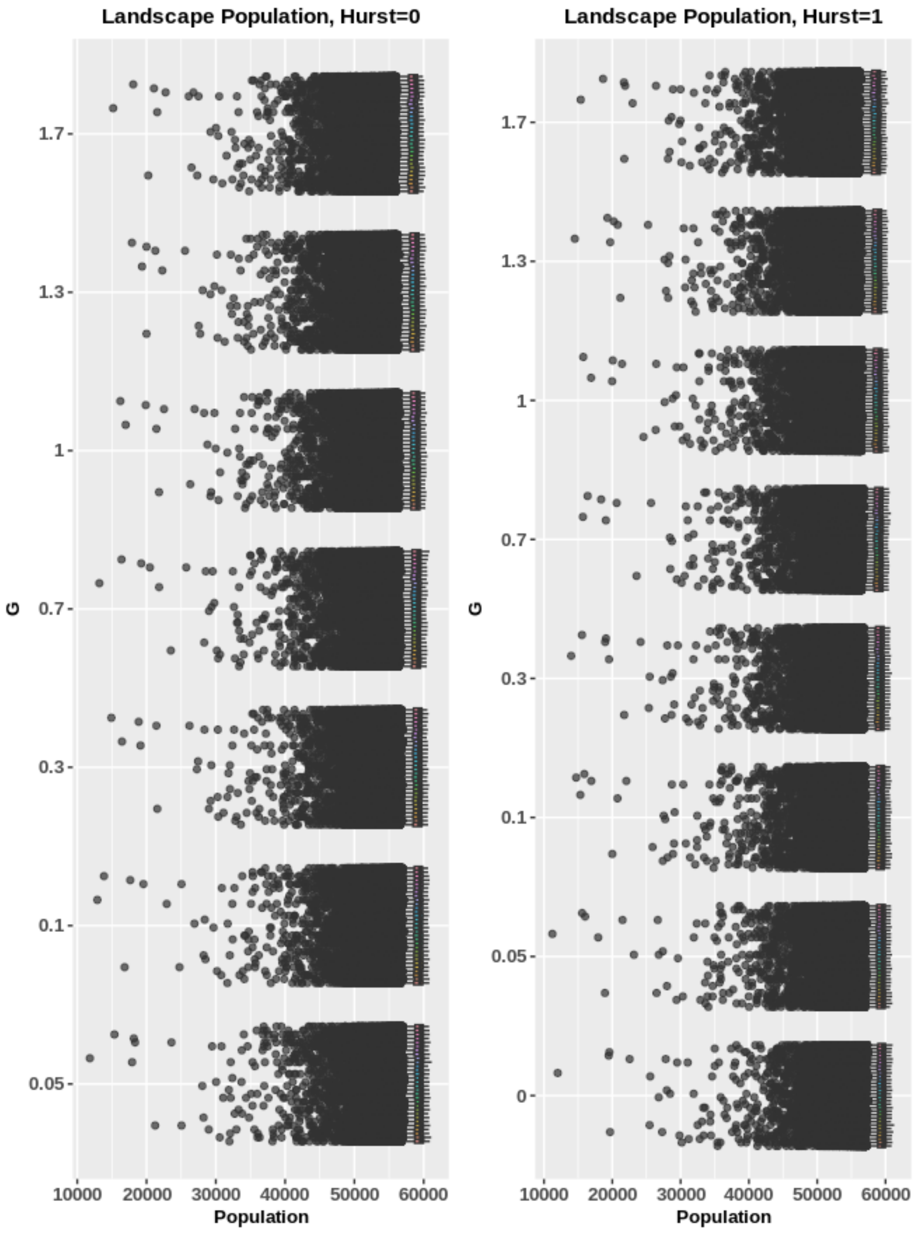

Supplementary Figure S5. Box plots of landscape total population for individual replicates by G and Hurst index.

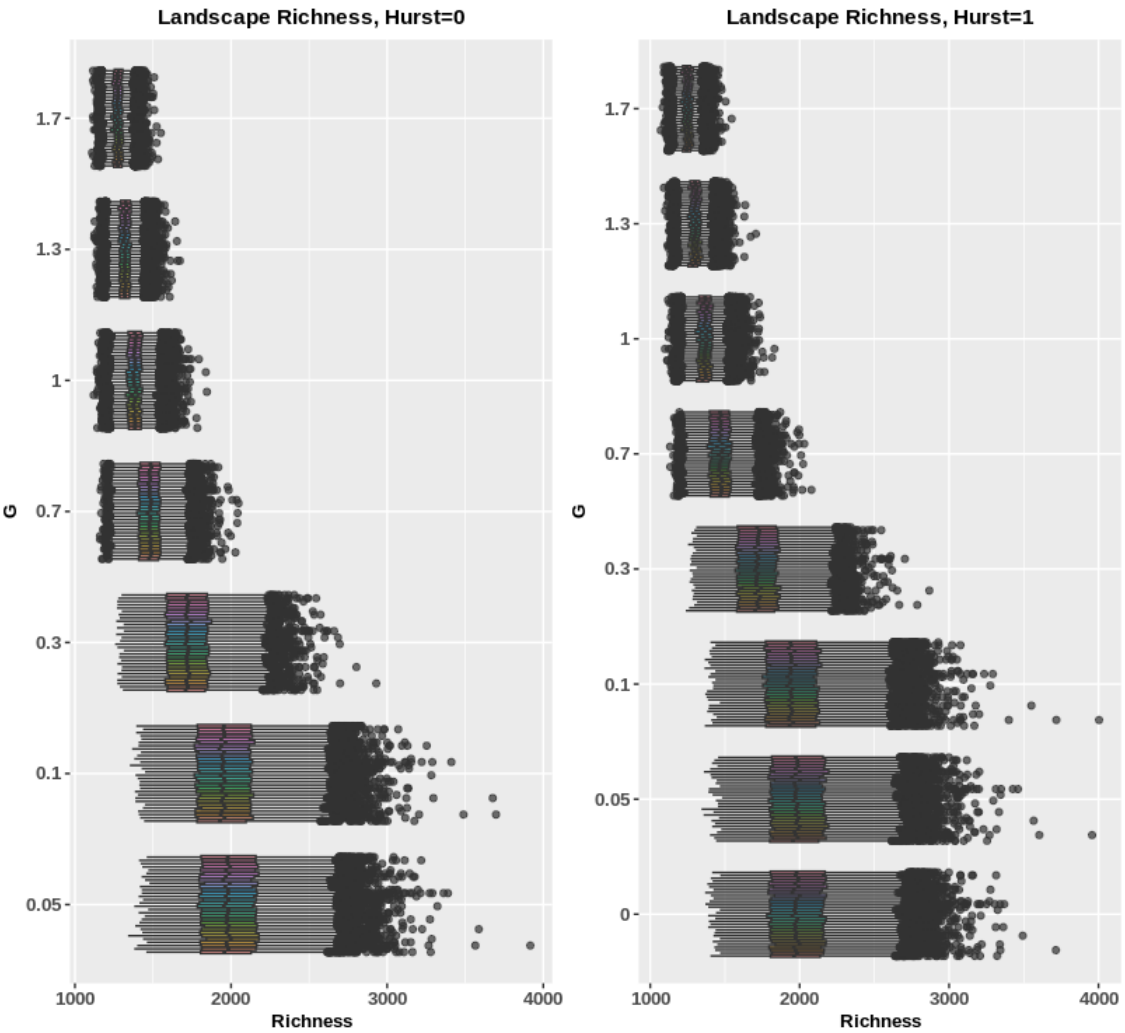

Supplementary Figure S6. Box plots of landscape richness for individual replicates by G and Hurst index.

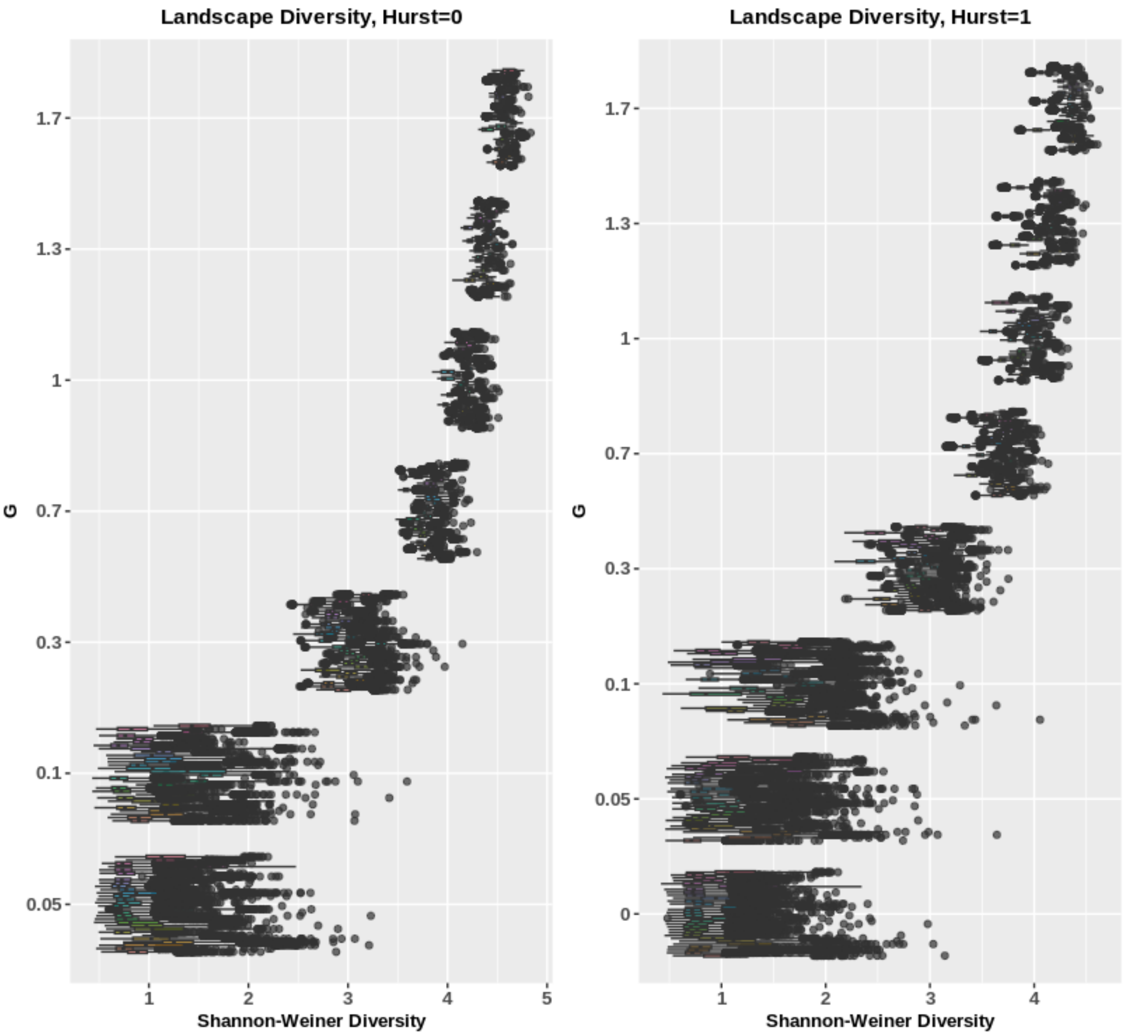

Supplementary Figure S7. Box plots of landscape Shannon-Wiener index for individual replicates by G and Hurst index.

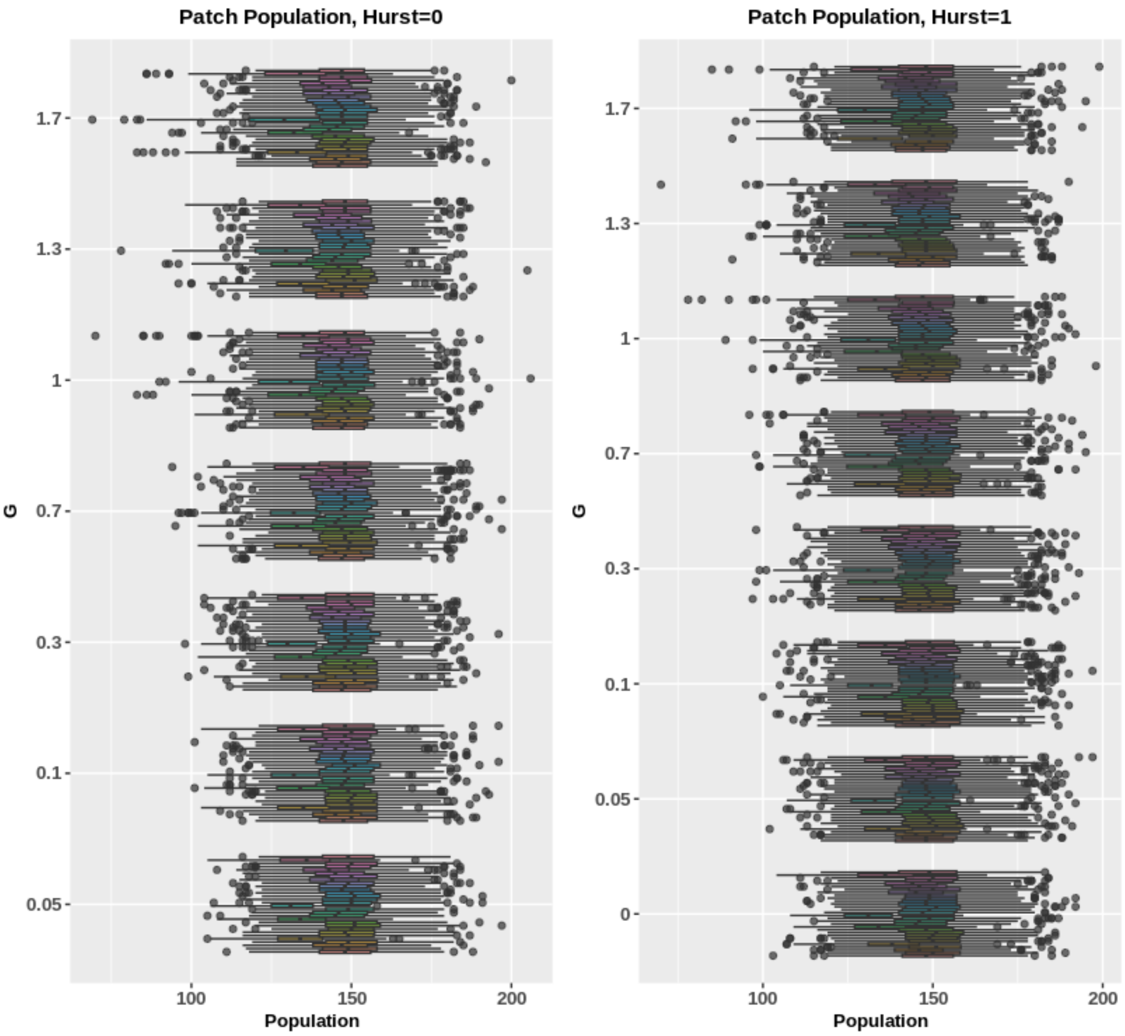

Supplementary Figure S8. Box plots of patch total population for individual replicates by G and Hurst index.

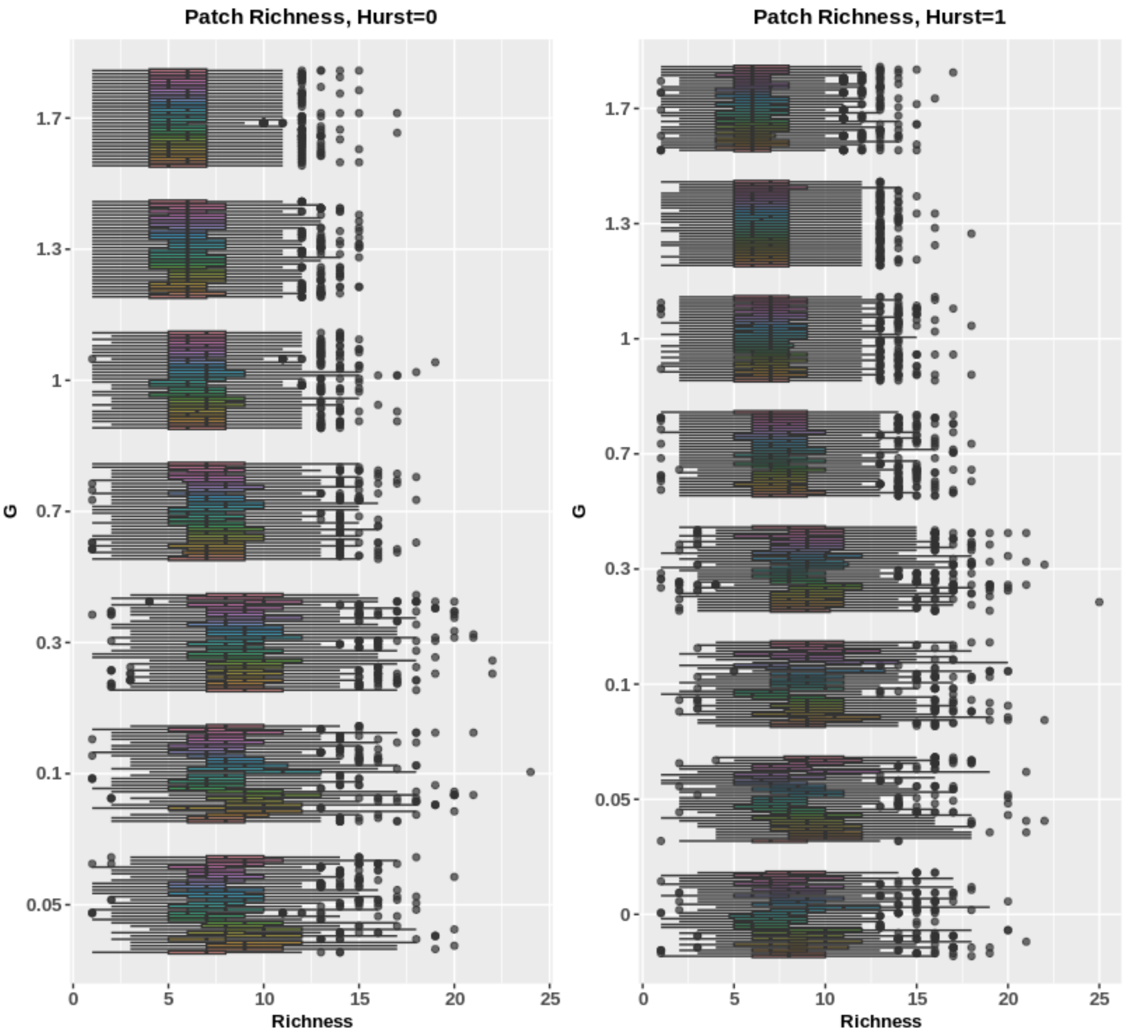

Supplementary Figure S9. Box plots of patch richness for individual replicates by G and Hurst index.

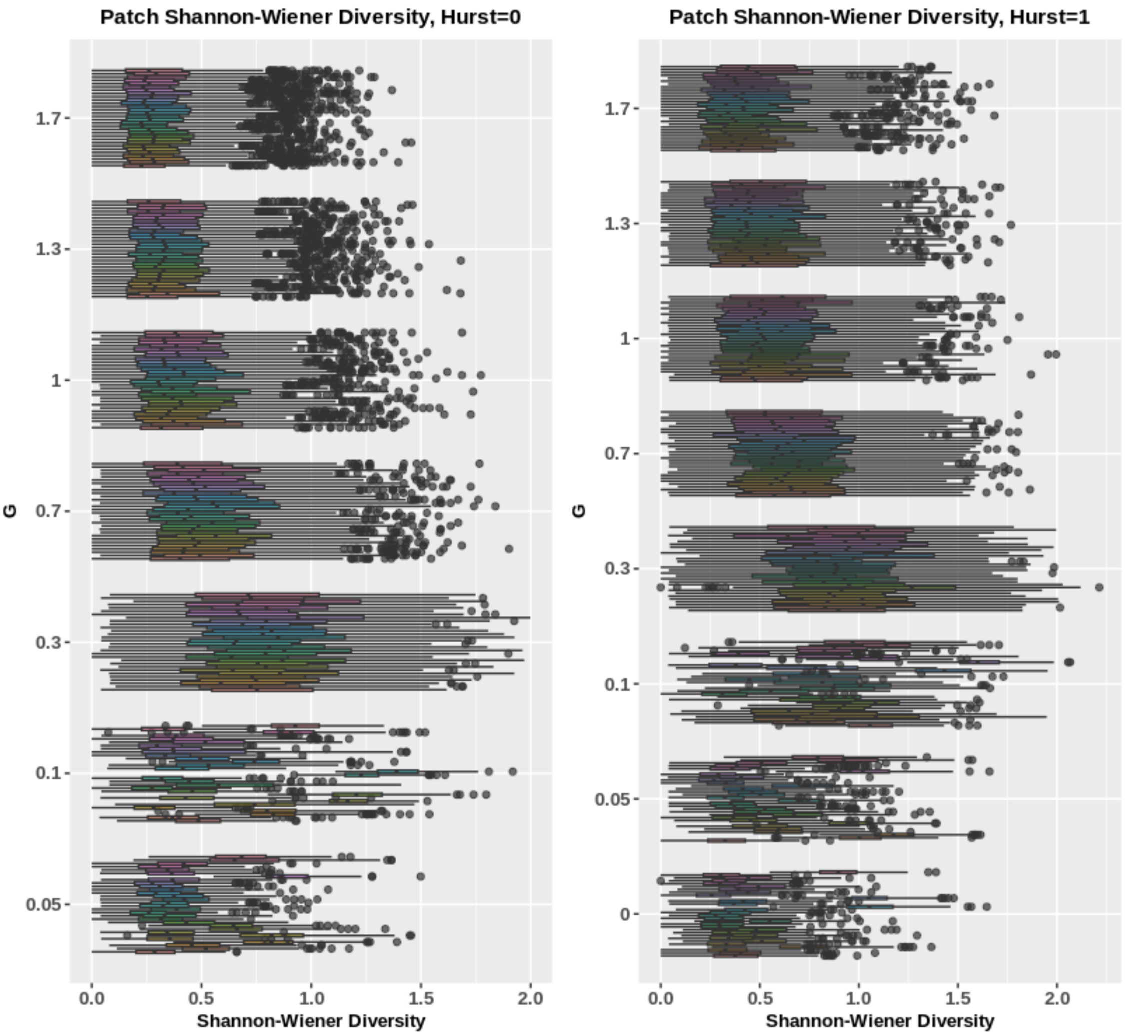

Supplementary Figure S10. Box plots of patch Shannon-Wiener index for individual replicates by G and Hurst index.
